# *In-situ* Adsorption-Biological Combined Technology Treating Sediment Phosphorus in all Fractions

**DOI:** 10.1038/srep29725

**Published:** 2016-07-15

**Authors:** Y. Zhang, C. Wang, F. He, B. Liu, D. Xu, S. Xia, Q. Zhou, Z. Wu

**Affiliations:** 1 State Key Laboratory of Freshwater Ecology and Biotechnology, Institute of Hydrobiology, Chinese Academy of Sciences, Wuhan 430072, China; 2 Graduate University of Chinese Academy of Sciences, Beijing 100039, China; 3 School of Resources and Environmental Engineering, Wuhan University of Technology, Wuhan 430070, China

## Abstract

The removal efficiency of sediment phosphorus (P) in all fractions with *in-situ* adsorption-biological combined technology was studied in West Lake, Hangzhou, China. The removal amounts of sediment Ca-P, Fe/Al-P, IP, OP and TP by the combined effect of PCFM (Porous ceramic filter media) and *V. spiralis* was 61 mg/kg, 249 mg/kg, 318 mg/kg, 85 mg/kg and 416 mg/kg, respectively, and the corresponding removing rate reached 10.5%, 44.6%, 27.5%, 30.6% and 29.2%. This study suggested that the combination of PCFM and *V. spiralis* could achieve a synergetic sediment P removal because the removal rates of the combinations were higher than the sum of that of PCFM and macrophytes used separately. From analysis of sediment microbial community and predicted function, we found that the combined PCFM and *V. spiralis* enhanced the function of P metabolism by increasing specific genus that belong to phylum Firmicutes and Nitrospirae. Thus it can be seen the *in-situ* adsorption-biological combined technology could be further applied to treat internal P loading in eutrophic waters.

Phosphorus (P) is one of the greatest concerns because it can contribute significantly to eutrophication in lakes^1^,^2^,^3^. The majority of freshwater lake restoration programmes are now striving towards an improvement in water quality through the reduction of external P loading^4^,^5^,^6^, while external P control is not always possible, especially if lake sediments are relevant^2^,^7^,^8^,^9^. Sediment is usually a major sink for Pin lakes, and contains a very large P pool in comparison to that in the water column. The exogenous phosphorus input will gradually be put under control by cutting-off of pollutant discharge and strengthening the ecological restoration. Upon controlling the input of external P loading, sediment plays a more important role in the P metabolism in lakes^10^,^11^. So the internal loading caused by P release from sediment under certain environmental conditions is the decisive factor for the P content in a lake, thus deciding the eutrophication status of water body.

Various remediation strategies have been developed to control internal P loading, such as precipitation of P by physicochemical methods^12^,^13^,^14^, sediment dredging^15^, *in-situ* physical adsorption methods and phytoremediation^16^, etc. Formation of a macrophyte-dominated state can create a positive feedback mechanism leading to self-stabilization of lake ecosystem at desired quality^17^,^18^. Many researches about submerged macrophytes reducing sediment resuspension and controlling the release of internal P loading have been reported in recent years^19^,^20^,^21^. While the treatment effect of submerged macrophytes on sediment P is often limited by the growing cycle and the surrounding environment, thus a single eco-technology can no longer be used effectively and universally, a combined technology is urgently needed to treat sediment P.

*In-situ* physico-chemical controls are potentially effective technologies to reduce the pollution of internal P, stabilize sediments and reduce dissolved contaminant transport into overlying waters^22^,^23^. To further enhance the effect of submerged macrophytes on sediment remediation, and make up the shortage of the single technology, the combined technology of physico-chemical and submerged macrophytes may be a highly effective way to control internal P loading.

The new porous ceramic filter media (PCFM) developed by means of red mud as main raw material, has the characteristics of anticorrosion, heat-resistant, high mechanical strength, no secondary pollution, long service life, large specific surface, low cost and high adsorption capacity. As a kind of ecological environment material, it has more extensive spreading values and prospects for applying^24^. In the paper, we choose PCFM as physico-chemical adsorbent, and the removal effect of sediment P in all fractions with the combined technology of PCFM and submerged macrophytes was conducted in West Lake, Hangzhou, China.

High throughput sequencing of 16 s rRNA amplicon was used to figure the microbial community changed by the combined technology. The key genera responsible for P metabolism was further predicted by PICRUSt (Phylogenetic Investigation of Communities by Reconstruction of Unobserved States) which was a newly developed software^25^ and already applied for functional prediction in wheat straw degrading microbial consortia^26^, streams with a gradient of alkaline mountaintop mine drainage^27^ and changes of root-zone microbial community by plant invasions^28^. This is the first time to use PICRUSt in removal mechanism of sediment P in all fractions with the combined technology of PCFM and submerged macrophytes.

## Results and Discussion

### Sediment characteristics

The general features and chemical component concentrations in West Lake sediments were presented in Table 1. The sediment was siliceous, and total proportions of SiO_2_, Al_2_O_3_,CaO, Fe_2_O_3_ and K_2_O were more than 80%. The grain size of the sediment was almost less than 100 mm. TOC content of the sediment was 9.82%. The content of TP was relatively high, reached 1424 mg/kg, Ca-P and Fe/Al-P was about 40.8% and 39.2% of TP, respectively, IP amounted to 80.5% of TP in the sediment, and OP accounted for about 19.5%.Thus, it can be seen that the sediment was unstable, at risk of releasing P to lake.

### P adsorption on PCFM by stirring tests

#### Effect of dosage of PCFM

**Figure 1:**
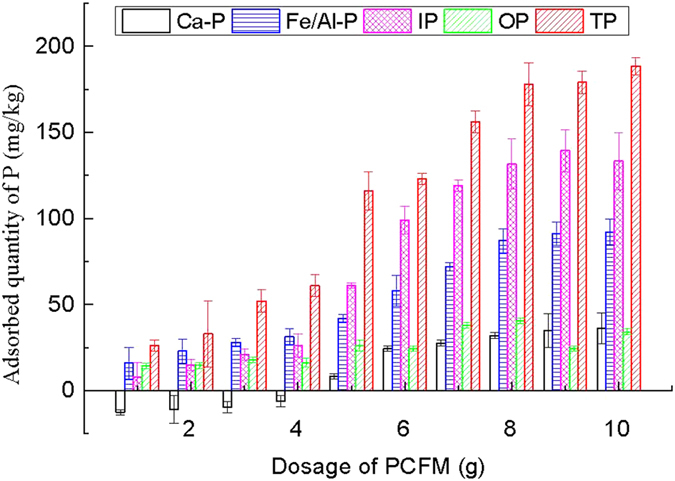
Effect of dosage of PCFM on adsorption of P in all fractions.

To investigate the effect of dosage on P adsorption from the sediment by PCFM, experiments were carried out with varying dose at 20 ± 2 °C and at a constant stirring speed of 200 rpm for 12 h, the results are presented in Fig. 1. In general, the increase in PCFM dosage increased the percent removal of P from the sediment compared with the control group. Ca-P appeared negative growth when PCFM dosage was less than 4 g, and the adsorption quantity of Ca-P gradually increased as the PCFM dosage was more than 4 g, it can be seen that a small amounts of PCFM can promote other P forms transformed to Ca-P. The increased trend of each P forms adsorption quantity become smooth when PCFM dosage was 8 g~10 g. The adsorption quantity of Ca-P, Fe/Al-P, IP, OP and TP (PCFM dosage was 8 g) was 32 mg/kg, 87 mg/kg, 132 mg/kg, 41 mg/kg and 178 mg/kg, respectively, the corresponding removal rate was 5.5%, 15.6%, 11.5%, 14.7% and 12.5%. The dosage of PCFM 8 g was chosen as optimal dosage to investigate the effect of other factors on the sorption capacity.

#### Effects of oscillating time

**Figure 2:**
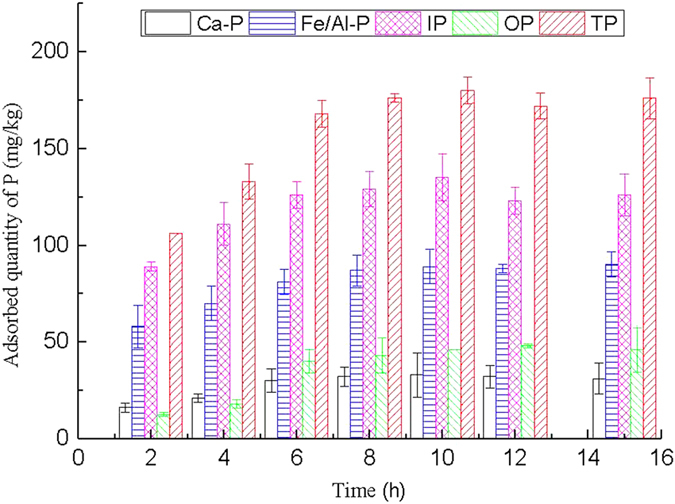
Effect of oscillating time on adsorption of P in all fractions.

The effect of oscillating time on P adsorption on PCFM at 20 ± 2 °C and at a constant stirring speed of 200 rpm is shown in Fig. 2. As seen from Fig. 2, the sorption of each P forms continued to proceed at a slow rate and equilibrium was reached within 8 h after the initial rapid adsorption process, and the adsorption quantity of each P forms decreased slightly as the oscillating time was 12 h, and then reached equilibrium. Thus, 8 h was selected as a consistent time to investigate the effect of pH and temperature on the sorption capacity.

#### Influence of operation temperature

**Figure 3:**
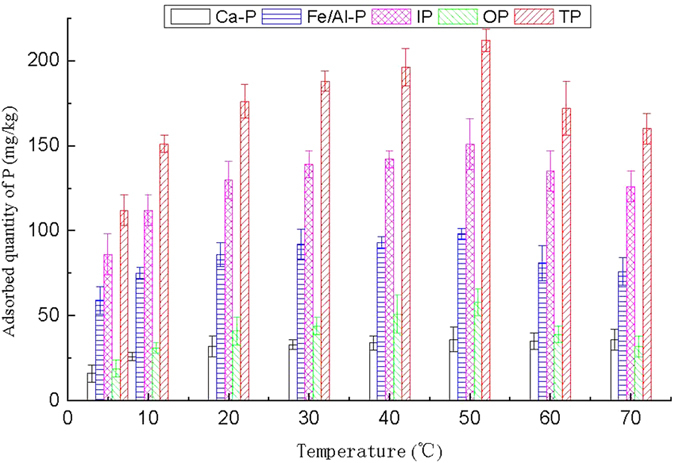
Effect of temperature on adsorption of P in all fractions.

Temperature is usually considered as an important factor for adsorption process^24^,^29^. The P adsorption from the sediment in West Lake by PCFM was studied at various temperatures ranging from 5 °C to 70 °C. The adsorbed quantities of each P forms in the sediment after the adsorptive reaction are illustrated in Fig. 3. When temperatures ranging from 5–50 °C, the amounts of P sorption increased with temperature rising. The adsorption quantity of Ca-P, Fe/Al-P, IP, OP and TP at 5 °C was 16 mg/kg, 59 mg/kg, 86 mg/kg, 19 mg/kg and 112 mg/kg, respectively, correspondingly, the removal rate was 2.8%, 10.6%, 8.2%, 6.8% and 7.9%, respectively. And the adsorption quantity of Ca-P, Fe/Al-P, IP, OP and TP on PCFM at 50 °C was 36 mg/kg, 98 mg/kg, 151 mg/kg, 58 mg/kg and 212 mg/kg, respectively, the corresponding removal rate was 6.2%, 17.6%, 13.2%, 20.9% and 14.9%, respectively. When operation temperature escalated above 50 °C, the adsorption amount of all P forms was decreased in varying degrees. It was speculated that the phenomena described above was attributed to the possibility that high temperatures increased sorption process and the Brownian movement was enhanced with temperature rising^30^.

#### Effects of pH on P adsorption

**Figure 4:**
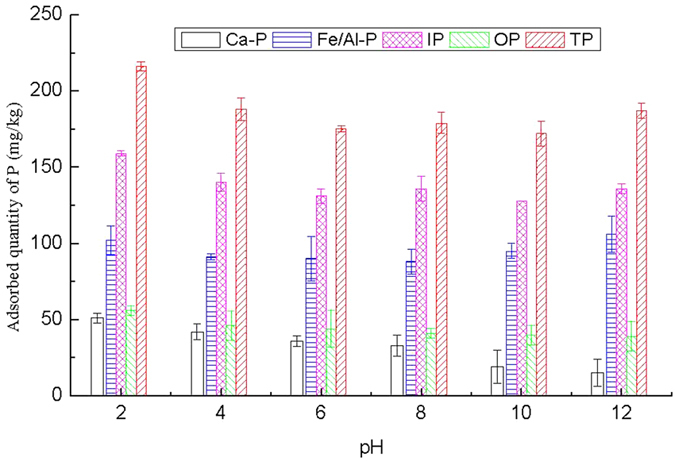
Effect of operation pH on adsorption of P in all fractions.

The effect of the operation pH on P adsorption from sediment was studied at pH 2–12. The experimental data are illustrated in Fig. 4. The adsorption efficiency of each P forms except Fe/Al-P was highest under the strong acid conditions, and both acid and alkalinity conditions promoted the adsorption of Fe/Al-P. The adsorption quantity of Ca-P, Fe/Al-P, IP, OP and TP in the sediment at pH 2 was 51 mg/kg, 102 mg/kg, 159 mg/kg, 56 mg/kg and 216 mg/kg, respectively. Correspondingly, the adsorption rate was up to 8.8%, 18.3%, 13.9%, 20.1% and 15.2%, respectively. The adsorption quantity of Ca-P, IP, OP and TP decreased in varying degrees when pH increased from 2 to 12, while the adsorption quantity of Fe/Al-P was the highest in alkaline conditions.

#### Static adsorption tests

**Figure 5:**
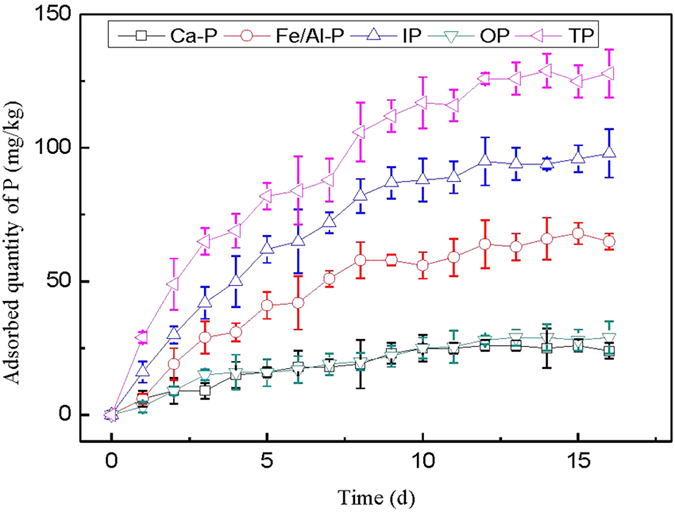
Effect of static adsorption time on P adsorption.

To simulate the *in-situ* treatment of sediment P in all fractions in West Lake sediment, static adsorption tests were conducted with adsorption time from 0 d to 16 d and the results are shown in Fig. 5. It can be seen that P adsorption on PCFM included quick, slow and dynamic balance adsorption steps, the quick adsorption step mainly occurred within 8 d, then followed by a slower second step (8–12 d). There was no apparent difference in adsorption amount of each P forms after 12 d, indicating that the adsorption-equilibrium occurred. The adsorption amounts of Ca-P, Fe/Al-P, IP, OP and TP on 12 d was 26 mg/kg, 64 mg/kg, 95 mg/kg, 28 mg/kg and 126 mg/kg, respectively. Correspondingly, the adsorption rate reached 4.5%, 11.5%, 8.3%, 10.1% and 8.8%, respectively. It is clear that the simple *in-situ* treatment method can reduce the sediment P in all fractions, thus control the internal P load in some extent.

### Combined effect of PCFM and submerged plants treating sediment P

#### Removal effect of sediment P by *V. spiralis*

**Figure 6:**
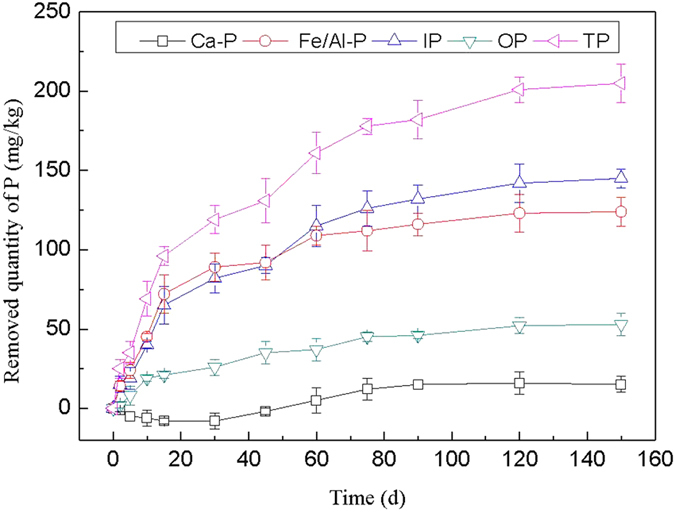
Removal effect of sediment P by *V. spiralis*

The removal effect of each P forms in sediment by *V. spiralis* is shown in Fig. 6. As seen from Fig. 6, the removal efficiency of Ca-P appeared negative growth in the first 45 d, then the adsorption quantity of Ca-P gradually increased, and the maximum removal amount of Ca-P was 16 mg/kg, the corresponding removal rate was 2.8%. It can be seen that other P forms transformed to Ca-P in the initial processing of *V. spiralis* treating sediment P. The removing rate of Fe/Al-P, IP, OP and TP increased rapidly within 45 d and then flattened, and reached equilibrium after 120 d. The removal amount of Fe/Al-P, IP, OP and TP by *V. spiralis* on 120 d was 123 mg/kg, 142 mg/kg, 52 mg/kg and 201 mg/kg, respectively, and the corresponding removing rate was 22.0%, 12.4%, 18.7% and 14.1%.

#### Effects of thickness of PCFM on the concentration of sediment TP

**Figure 7:**
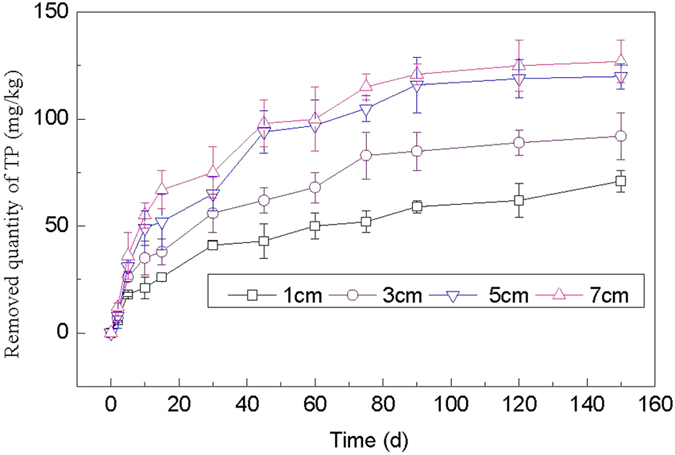
Effect of thickness of PCFM on P removal.

To investigate the optimal thickness of PCFM on P adsorption from the sediment, experiments were carried out with varying thickness (1 cm, 3 cm, 5 cm and 7 cm), the results are presented in Fig. 7. The adsorption quantity of sediment TP on 90 d was 116 mg/kg as the thickness was 5 cm, the corresponding removal rate was 8.1%, and from then on, the adsorption quantity had no obvious change. The adsorption quantity of TP on 150 d added just 11 mg/kg when the thickness increased to 7 cm, and the corresponding removal rate was only added 0.8%. Synthesized the removal effect and economic cost, the thickness of PCFM 5 cm was chosen as optimal dosage to investigate the combined effect of PCFM and submerged plants on sediment P in West Lake.

#### Effects of combined effect of PCFM and *V. spiralis*

**Figure 8:**
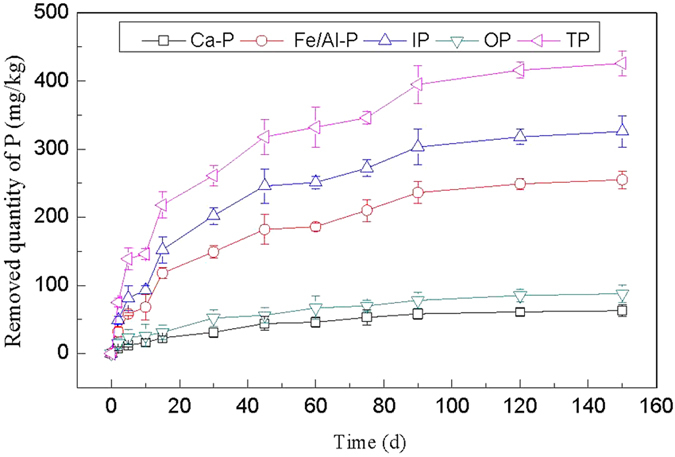
Combined effect of PCFM and *V. spiralis* on sediment P.

Effect of combined effect of PCFM and *V. spiralis* on each sediment P forms is shown in Fig. 8. As seen from Fig. 8, the removing rate of each sediment P forms increased obviously within 45 d and flattened after 90 d. The removal amount of Ca-P, Fe/Al-P, IP, OP and TP on 120 d by the combined effect of PCFM and *V. spiralis* was 61 mg/kg, 249 mg/kg, 318 mg/kg, 85 mg/kg and 416 mg/kg, respectively, and the corresponding removing rate was 10.5%, 44.6%, 27.5%, 30.6% and 29.2%. The results showed that Ca-P get the lowest removal amount and Fe/Al-P get the highest one, the potential reason was that: Ca-P was HCl-extractable P, P associated with Ca, Ca-P mainly includes apatite P generated by authigenic or biogenic, and calcium phosphate coprecipitated with authigenic calcium carbonate or exogenous input of insoluble calcium phosphate minerals, such as hydroxyapatite, calcium superphosphate, etc. Such P form with high chemical stability is difficult to be adsorbed on adsorbent, and difficult to be utilized by microorganism and aquatic plant. Fe/Al-P was NaOH-extractable P, P bound to Al, Fe and Mn oxides and hydroxides. The transport-transformation process of Fe/Al-P was one of the main mechanisms for P releasing to overlying water from sediment, it is the main reactive P component in sediment. Thus, Fe/Al-P was relatively easy to be adsorbed on PCFM and utilized by *V. spiralis* and microorganism.

**Figure 9:**
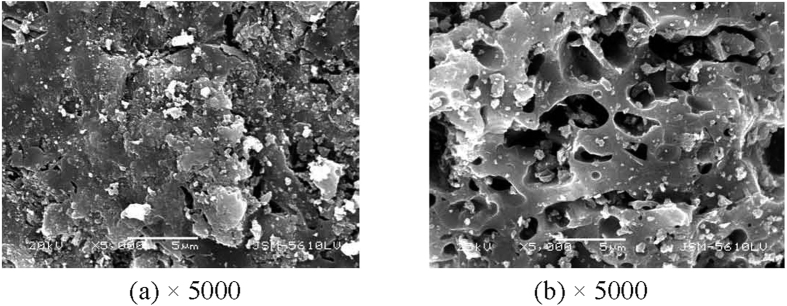
SEM photographs of PCFM ((**a**) surface, (**b**) cross section).

**Figure 10:**
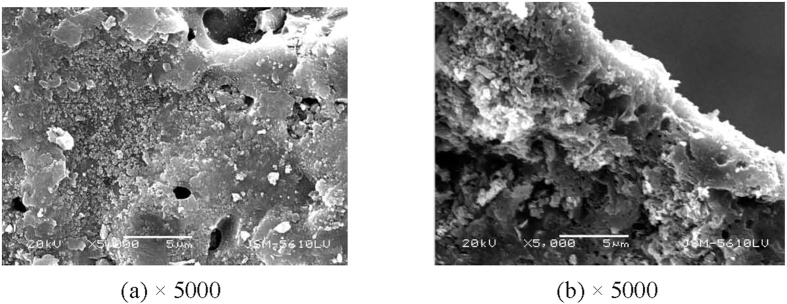
SEM photographs of PCFM after the combined adsorption-biological process ((**a**) surface, (**b**) cross section).

Table 2 showed the maximum removal amount of sediment TP with the three treatments, we can find that the combination of PCFM and *V. spiralis* have more significant effect on sedimental P removal than the sum effect of single PCFM and *V. spiralis*. Figures 9 and 10 showed the SEM photographs of PCFM before and after the combined adsorption-biological process. Compared Fig. 9 with Fig. 10, we found that a layer of thin film deposited on the surface of PCFM, which might be biofilm, thus microbial community and predicted function test was conducted in the latter part.

#### Microbial community and predicted function

**Figure 11:**
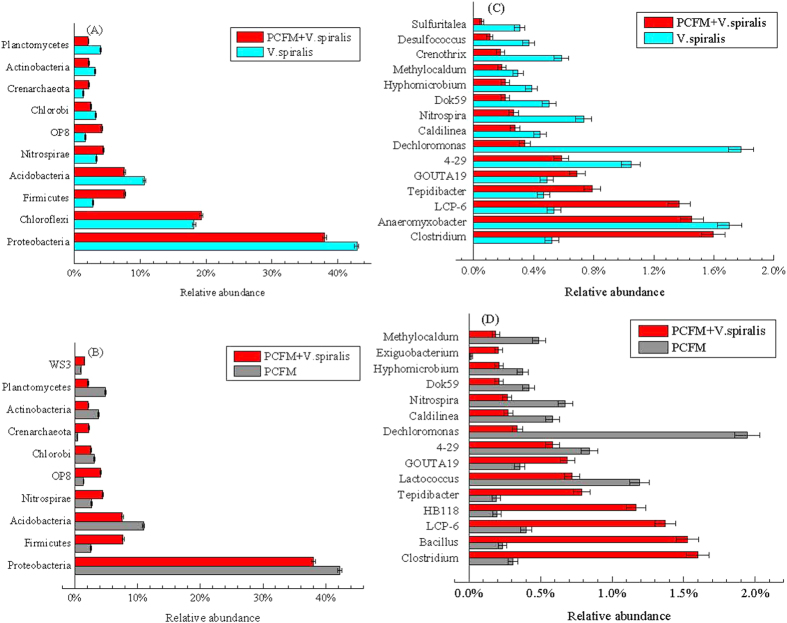
Comparison analysis of microorganisms with significant difference (P < 0.05) between different treatments (PCFM vs PCFM + *V. spiralis*; PCFM vs PCFM + *V. spiralis*) on phylum (**A,B**) and genus (**C,D**) level.

After aligning the OTUs with databases mentioned in the method, we classified the sediment microbial community into phyla and genera. Paired comparisons of each relative abundance were applied between the PCFM and the combined and between the *V. spiralis* and the combined. Classifications with significant difference (P < 0.05) were showed in Fig. 11. At phylum level, Firmicutes, Nitrospirae, OP8 and Crenarchaeota were increased, while Proteobacteria, Acidobacteria, Chlorobi, Actinobacteria and Planctomycetes were decreased in the combine group compared with either PCFM or *V. spiralis*. At genus level, *Clostridium*, *Tepidibacter*, *LCP-6* and *GOUTA19* were increased, while *Nitrospira*, *4–29*, *Dechloromonas*, *Dok59*, *Hyphomicrobium*, *Methylocaldum* and *Caldilinea* were decreased in the combine group compared with either PCFM or *V. spiralis*. The increases of genera *Bacillus*, *Exiguobacterium*, *HB118* were specific in combined group compared with PCFM group, while the decreases of genera *Anaeromyxobacter*, *Crenothrix*, *Desulfococcus* and *Sulfuritalea* were specific in combined group compared with *V. spiralis* group.

**Figure 12:**
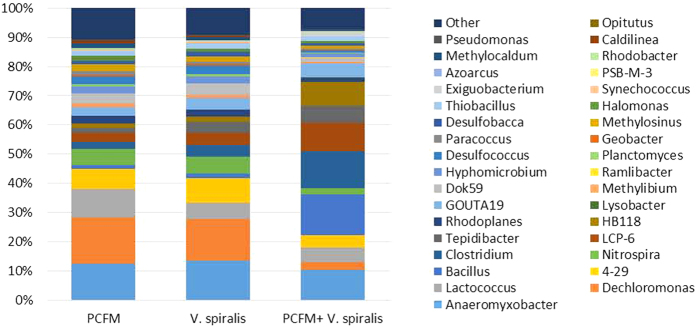
Contributions of each genus (top 35) that involved in P metabolism function estimated by PICRUSt.

Relative gene family abundances were generated by the predicted functional profile using PICRUSt and grouped into P metabolism functional categories. Sum of relative abundance of 359 KOs involved in P metabolism were 9.59%, 9.65% and 9.73% in the treatment group of PCFM, *V. spiralis* and PCFM +*V. spiralis*, respectively, which indicated a more active P transformation in the combine group than in other two. OUTs at genus level that contributed to P metabolism were showed in Fig. 12. Genus *Anaeromyxobacter* in phylum Proteobacteria, *Clostridium* and *Bacillus*in Firmicutes, *HB118* and *LCP-6* in Nitrospirae were the major contributors involved in P. These genera were also included in the significantly difference genera list that shown in Fig. 12. Except for *Anaeromyxobacter*, other genera of the major contributors were all increased in the combine group compared with the other two group. Therefore, the combined PCFM and *V. spiralis* enhanced the function of phosphorus metabolism by increasing specific genus that belong to phylum Firmicutes and Nitrospirae.

*Bacillus sp.* has been used asphosphate-solubilizing in agriculture soil^31^ and showed excess poly-P accumulation serves as P storage for sediment bacteria^32^. Aerobic/extended-idle biological phosphorus removal processes was dominated by *Clostridium spp*.^33^. *Exiguobacterium* that was one of the increased genus specific in combined group compared with PCFM group, has been isolated asphosphate solubilizing bacteria and are reported to increase growth and biomass of *Vigna unguiculata*^34^. Species belonged to genera *Bacillus* and *Clostridium* also have the ability to secrete alkaline phosphatase^35^, activity of which could be enhanced by submerged macrophyte and its rhizospheric microbial community and further increase soluble reactive P in sediment^36^. Microbes belongs to these specific genus all existed in the PCFM group and *V. spiralis* group, but they increased obviously n the combined group under the interaction of PCFM and *V. spiralis*. The flourish of P-solubilizing microbes might be originally attributed to plant P deficiency that caused by quickly PCFM absorption, then root exudation that directly for P-solubilizing or favors bioavailable P producing microbes was stimulated as plant biological response^37^. Thus, the increased amount of bioavailable P promote P absorption on PCFM and the growth of submerged macrophytes (Table 3), which enhanced further P utilization by submerged macrophytes. *V. spiralis* changed the residual P forms in the sediment not adsorbed on PCFM through shoot oxygenation and nutrition allocation, then increase the extra P adsorption on PCFM.

## Conclusions

The study demonstrated the treatment effect of sediment P in all fractions in West Lake with combined technology of PCFM and submerged macrophyte. The major results can be summarized as follows:

1. PCFM dosage, reaction time, operation temperature and pH were the main factors influencing the sediment P adsorption on PCFM.
2. The removal quantity of Ca-P, Fe/Al-P, IP, OP and TP from the sediment under static adsorption tests reached 26 mg/kg, 64 mg/kg, 95 mg/kg, 28 mg/kg and 126 mg/kg, respectively.
3. The removal amounts of Ca-P, Fe/Al-P, IP, OP and TP by the combined effect of PCFM and *V. spiralis* was 61 mg/kg, 249 mg/kg, 318 mg/kg, 85 mg/kg and 416 mg/kg, respectively. The combination of PCFM and macrophyte could achieve a synergetic sediment P removal because the removal rates of the combinations were higher than the sum of that of PCFM and macrophyte used separately.
4. Specific genera that belong to phylum Firmicutes and Nitrospirae were the key genera which responsible for the synergetic sediment P removal effect under the combination of PCFM and *V. spiralis*.

## Methods

### Study sites and sampling

West Lake (120°08′E, 30°15′N) is a famous resort lake, as well as a typical shallow and eutrophic lake, listed in the World Heritage Site in 2011, located on the western side of Hangzhou City, China. Having a water area of about 6.5 km^2^, average depth of 2.27 m, West Lake is 3.2 km from north to south, 2.8 km from east to west.

Sampling site is located in Xiaonan Lake (one son lake of West Lake) in the west of the lake, a severe eutrophic region (30°23′16′′N, 120°13′18′′E). Surface sediment samples (0–10 cm) were collected using a Peterson sediment collector (model HNM1-2, with a surface area of 0.1 m^2^). The samples were taken to laboratory in air-sealed plastic bags and kept at 4 °C. Then samples were freeze-dried and sieved with a standard 80 mesh to remove big particles for further experiments. Water samples were collected 0.5 m below the water surface and were immediately filtered through 0.45 mm cellulose acetate membranes.

**Figure 13:**
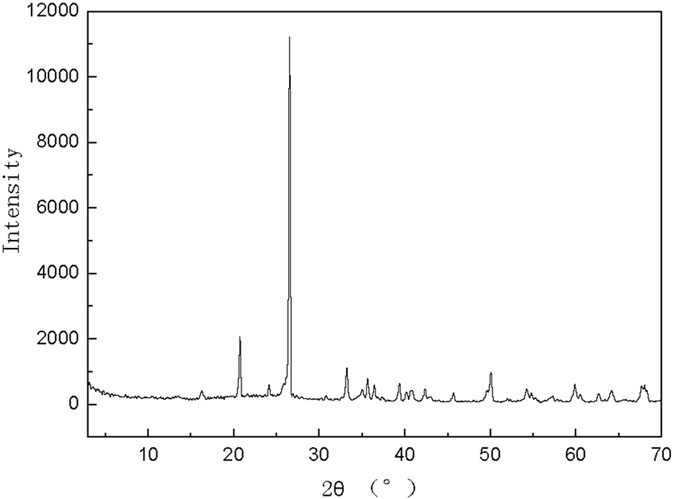
XRD patterns of PCFM.

The PCFM used in this study was obtained from Shandong Aluminium Industry Corporation, Shandong province, China. The chemical compositions of the PCFM are shown in Table 4. It consists primarily of SiO_2_, Al_2_O_3_ and Fe_2_O_3_. In addition, it contains Ba, Cu, Li, Mn, V, Zn and a small amount of alkaline-earth metals oxides (Fig. 13). The PCFM has large porosity and specific surface with rough surface and porous interior (Fig. 9). The BET surface area of PCFM with particle size of 3–5 mm was 4.92–4.98 m^2^/g, the apparent density of PCFM was 1.02–1.19 g/cm^3^ and the porosity was 55%.

### Batch experiments

#### Adsorption experiments

Stirring experiments were performed in vessels, containing 250 mL KCl solution, a certain quantity of PCFM and 5 g sediment samples shaken for some time at different pH and temperature at 200 rpm. Two drops of 0.1% chloroform was added to inhibit bacterial activity. Then the P concentration in the sediment was determined after the adsorption process. Static experiments were conducted in vessels under static condition with adsorption time from 0 d to 16 d.

#### Phytoremediation experiments

Submerged plant *Vallisneria spiralis* (*V. spiralis*) was collected from the West Lake. Samples were rinsed to remove invertebrate grazers and first pre-cultured in the experimental plot for 7 d. Then, the plants (approximately 20 cm in length) were transplanted to the polythene buckets (diameter 60 cm, height 80 cm). A layer of sediment with 10 cm in thickness was placed in each bucket. The water level was maintained at 60 cm height throughout the experimental period (macrophyte group). Sediment and water filled in were all taken from Xiaonan Lake, Hangzhou City.

#### Combined experiments

Submerged plants (*V. spiralis*) (approximately 20 cm in length) were transplanted to the polythene buckets (diameter 60 cm, height 80 cm). A layer of sediment with 10 cm in thickness was placed in each bucket, then a layer of PCFM with certain thickness was placed on the sediment (combined group). The experiments without macrophyte was set as PCFM group. The water level was maintained at 60 cm height throughout the experimental period.

#### Microcosm experiments

Polythene buckets (diameter 60 cm, height 80 cm) filled with sediment in 10 cm thickness and water in 60 cm height were used to make microcosms. Sediment and water were all taken from Xiaonan Lake, Hangzhou City. Submerged plant (*V. spiralis*) approximately 20 cm in length was collected from the West Lake. They were rinsed to remove invertebrate grazers and pre-cultured in the experimental plot for 7 d. There were six treatments for microcosm experiments: with *V. spiralis* and without PCFM, without *V. spiralis* and with PCFM 1, 3, 5, 7 cm thickness respectively, with *V. spiralis* and with PCFM 5 cm. The microcosm experiments were performed over 150 days, during which water level was maintained at 60 cm height.

#### High throughput sequencing for sediment bacterial community

Sediment sub-samples were taken from the macrophyte group, PCFM group and the combined group on 150 d and stored at −80 °C for future use. DNA was extracted from sediment followed the protocol of the E.Z.N.A.™ Soil DNA Kit (Omega, USA).The bacterial hypervariable regions V4-V5 of the 16 S rRNA gene were PCR-amplified using individually bar-coded forward primers 515F, 5′-GTGCCAGCMGCCGCGG-3′ and reverse primer 907R, 5′-CCGTCAATTCMTTTRAGTTT-3′. Amplicon sequencing was carried out on the Illumina HiSeq 2500 platform to generate the 250 bp PE (pair end) raw reads. Raw reads were processed by the downstream steps: assigned to a sample by unique barcodes, paired-end reads were merged using FLASH (Magoc and Salzberg 2011), quality filtering were then performed under specific filtering conditions of QIIME^38^, chimeric sequences deletion using UCHIME algorithm^39^. Effective sequence number obtained from the above steps was 44884 on average. Operational taxonomic units (OTUs) were defined at 97% sequence similarity and annotated using the RDP (Ribosomal Database Project) classifier with database Green genes. Species relative abundance for each taxonomy levels were constructed using R software.2.5 Predictive functional profiling of sediment microbial communities.

To predict functional responses of the PCFM, *V. spiralis* and the combined group from our 16 S rRNA marker gene sequences, software PICRUSt (Phylogenetic Investigation of Communities by Reconstruction of Unobserved States; http://picrust.github.com) was used to generate a functional profile. Suggested methods for OTU picking with Green genes 13.5 was taken using Galaxy (http://huttenhower.sph.harvard.edu/galaxy/). Predicted gene family abundances were corrected for expected 16S rRNA gene copy number 1000 and analyzed in the functional classification scheme KEGG Orthology (KO). KOs involved in phosphorus metabolism pathway were picked out to obtain the total functional abundance in each sample. Ways that determined which OTUs are contributing to phosphorus metabolism function were followed by the description on website (http://picrust.github.io/picrust/scripts/metagenome_contributions.html#metagenome-contributions).

#### Analytical methods

P fractions were determined using the SMT harmonized protocol^40^. Each P fractions was measured using the ammonium molybdate spectrophotometric method with UV-visible spectrophotometer (DR4000/U, HACH company, USA). The pH of the sediment was measured in 1:10 (w/v) solid/water suspensions with a pH meter (PHS-3C, Shanghai LeiCi instrument plant, China). The micrographs of PCFM were observed using a analytical transmission electron microscope (H-600 STEM/EDX PV9100, HITACHI, Japan). The compositions of PCFM and sediment were measured with the full spectrum of direct reading inductively coupled plasma emission spectrometer (Optima 4300DV, Perkin Elmer Ltd., USA).

All the chemicals and reagents used in this study were of analytical grade. All glassware and sample bottles were soaked in diluted HCl solution for 12 h, then washed and rinsed three times with deionized water. Deionized water was also used for the preparation of solutions. All experiment treatments were conducted in triplicate and all the data in the figures and tables are shown with standard deviation (SD).

## Additional Information

**How to cite this article**: Zhang, Y. *et al.* *In-situ* Adsorption-Biological Combined Technology Treating Sediment Phosphorus in all Fractions. *Sci. Rep.* **6**, 29725; doi: 10.1038/srep29725 (2016).

## Figures and Tables

**Table 1:** Properties and chemical compositions of sediment.

Properties		Content
Grain size (%)	<1 μm	5.91 ± 0.04
	1–10 μm	30.74 ± 0.25
	10–100 μm	60.98 ± 0.66
Major element (*wt.*%)	SiO_2_	54.68 ± 0.51
	Al_2_O_3_	15.15 ± 0.24
	CaO	4.95 ± 0.06
	Fe_2_O_3_	3.96 ± 0.05
	K_2_O	1.73 ± 0.02
pH		7.89 ± 0.09
TOC (%)		9.82 ± 0.10
P fractions (mg/kg)	Ca-P	581 ± 10.09
	Fe/Al-P	558 ± 10.18
	IP	1146 ± 18.24
	OP	278 ± 7.52
	TP	1424 ± 21.26

**Table 2:** The maximum removal amount of sediment TP with three treatments (mg/kg).

	PCFM	*V. spiralis*	PCFM and *V. spiralis*
TP	116 ± 5.3	201 ± 10.5	416 ± 22.2

**Table 3:** The *V. spiralis* biomass on 120 d (g/m^2^).

	*V. spiralis*	PCFM and *V. spiralis*
Biomass (stem leaf)	1156 ± 16.6	1694 ± 22.3
Biomass (root segment)	252 ± 6.1	381 ± 6.8

**Table 4:** The chemical compositions of PCFM (wt.%).

Compositions	SiO_2_	Al_2_O_3_	Fe_2_O_3_	CaO	Na_2_O	MgO	K_2_O
Content	38.25	21.96	18.07	8.64	9.14	2.90	0.52
